# In vivo study of the immune response to bioengineered spider silk spheres

**DOI:** 10.1038/s41598-022-17637-7

**Published:** 2022-08-05

**Authors:** Tomasz Deptuch, Karolina Penderecka, Mariusz Kaczmarek, Sara Molenda, Hanna Dams-Kozlowska

**Affiliations:** 1 Department of Cancer Immunology, Poznan University of Medical Sciences, 15 Garbary St, 61-866 Poznan, Poland; 2 Department of Diagnostics and Cancer Immunology, Greater Poland Cancer Centre, 15 Garbary St, 61-866 Poznan, Poland

**Keywords:** Nanomedicine, Nanotoxicology, Biomaterials, Nanobiotechnology

## Abstract

Bioengineered MS1 silk is derived from major ampullate spidroin 1 (MaSp1) from the spider *Nephila clavipes*. The MS1 silk was functionalized with the H2.1 peptide to target Her2-overexpressing cancer cells. The immunogenic potential of drug carriers made from MS1-type silks was investigated. The silk spheres were administered to healthy mice, and then (i) the phenotypes of the immune cells that infiltrated the Matrigel plugs containing spheres (implanted subcutaneously), (ii) the presence of silk-specific antibodies (after two intravenous injections of the spheres), (iii) the splenocyte phenotypes and their activity after restimulation ex vivo in terms of proliferation and cytokine secretion (after single intravenous injection of the spheres) were analyzed. Although the immunogenicity of MS1 particles was minor, the H2.1MS1 spheres attracted higher levels of B lymphocytes, induced a higher anti-silk antibody titer, and, after ex vivo restimulation, caused the activation of splenocytes to proliferate and express more IFN-γ and IL-10 compared with the PBS and MS1 groups. Although the H2.1MS1 spheres triggered a certain degree of an immunological response, multiple injections (up to six times) neither hampered the carrier-dependent specific drug delivery nor induced toxicity, as previously indicated in a mouse breast cancer model. Both findings indicate that a drug delivery system based on MS1-type silk has great potential for the treatment of cancer and other conditions.

## Introduction

Despite a large number of studies regarding nanosized drug delivery systems (DDSs), the interactions between nanoparticles (NPs) and organisms are not yet fully understood. The effects that NPs exert on organisms and their fate in the body are closely related to their physicochemical properties (e.g., the material used for production of the carrier, and their size, shape, surface chemistry, and biodegradability)^1^. NPs are mostly designed for systemic administration. After intravenous injection, the NPs directly interact with blood components^2^. As NPs enter the bloodstream, plasma proteins may adsorb on their surface, forming distinct protein coronas^3,4^. Opsonization of the nanoparticles may lead to their clearance from the body by mononuclear phagocytic cells (MPS) and, as a result, inhibit the efficient deposition of the carriers at the destination site^5^. Moreover, the administration of NPs may trigger the immune system^2^.

The immunostimulatory effects of NPs can be related to innate or/and adaptive immune responses^2^. The innate immune response may induce severe inflammatory reactions, complement activation-related pseudoallergies (CARPAs), or cytokine release storms (CRSs)^2^. Activated T and B lymphocytes are components of the specific adaptive immune response. B lymphocytes may produce antibodies capable of recognizing and binding NP antigens. The adaptive immune response is often noticeable in studies on lipid-based and polymeric NPs^6–8^. Some NPs can also act as haptens, and a nanoparticle-specific antibody response occurs only when the NPs are conjugated with other molecules. It has been reported that fullerene and dendrimeric carriers elicit the production of NP-specific antibodies when conjugated with bovine serum albumin (BSA), whereas no such response was observed in other studies regarding those types of carriers without BSA conjugation^9^.

Another major concern regarding the application of NP-based DDSs is their potential accumulation in organs, which may lead to long-term toxicity. Although many factors influence the NPs performance in the body, it is implicit that the size significantly affects their clearance as well as distribution. When the particle size exceeds 100 nm, the NPs may pass from the blood through fenestrae in the endothelium of liver, spleen, bone marrow (diameter up to 200 nm), and the kidney (fenestrae of 20–30 nm). Thus intravenously administrated NPs may be detected in blood and organs like liver, spleen, lungs, and kidney^10,11^. The toxicity issue may be especially associated with nonbiodegradable nanoparticles. To overcome this problem, biopolymeric carriers have gained much attention in recent years, as they often offer not only superior biocompatibility but also biodegradability. Silk is a fine example of such a biopolymer^12^. Nano- and microparticles made of silk fibroin originating from *Bombyx mori* or bioengineered spider silks can be employed as carriers for the delivery of drugs^13–17^. The major advantages of this material are its noncytotoxicity, good biodegradability, and its production of only mild immunogenic responses or no immunogenic response at all^18–20^.

Despite a large number of publications regarding the potential use of silk materials, there are few studies that address immune responses to silk materials in detail^19–23^. Furthermore, the majority of these studies relate to products made from regenerated silkworm silk, and only a few studies have described the immune response to materials made of recombinant silk^22^. Most of the currently available data concern the in vivo responses to an implantable silk material used for tissue regeneration (e.g., fibers^22^, films^19,21^, and scaffolds^23^). It has been reported that some of the implantable silk materials induced macrophage activation, a mild proinflammatory response, and a foreign body response^18,24^. An antibody-dependent response has been indicated for virgin silks, with the immune response triggered by sericins, which coating the silk fibroin and function as an adhesive^25^. Currently, to prevent immune activation, most silkworm silk processing techniques involve the removal of sericins during the degumming process. Unfortunately, because of the heterogeneity of the silk materials (i.e., silk sources, various processing methods, morphologies), the obtained data cannot be directly translated between studies; therefore, each material made of silk should be separately characterized.

In our previous work, we developed a drug delivery system based on MS1 bioengineered spider silk and its functionalized variant H2.1MS1^26^. The MS1 silk was based on the sequence of major ampullate spidroin 1 (MaSp1) from the spider *Nephila clavipes*. H2.1MS1 silk possesses an additional peptide sequence (H2.1) that selectively recognizes and binds to the Her2 receptor^26^. The Her2 receptor is overexpressed in several cancer types, including breast cancer, which makes it a good target for therapy. We performed the detailed characterization of the H2.1MS1 and control MS1 nanoparticles in terms of structure, morphology, size, zeta potential, stability, and drug loading/release capacity^17,26–28^. The MS1 and H2.1MS1 silks at a concentration of 0.5 mg/mL formed spheres whose size is approximately 400 nm in diameter, and their zeta potential is approximately 6 and 15 mV, respectively^17,27,28^. Moreover, nanospheres formed from the MS1 and H2.1MS1 silks were evaluated for their efficacy as drug delivery systems in both in vitro^26^ and in vivo studies^29^. The H2.1 functionalization of silk spheres allowed for the selective delivery of doxorubicin (Dox) to Her2-positive breast cancer cells^26^. Moreover, doxorubicin delivered by only functionalized H2.1MS1 particles selectively inhibited Her2-positive cancer growth in primary and metastatic mouse models^29^. Furthermore, in vivo toxicological studies showed that both types of spheres (MS1 and H2.1MS1) were nontoxic^30^. Although after 24 h, the intravenously (i.v.) administered silk nanoparticles showed their accumulation mainly in the liver and less in the lungs and spleen, the long-term biodistribution studies (up to 5 days) indicted the loss of the fluorescent signal of the nanospheres suggesting their clearance or/and possible degradation^30^. Moreover, 20 days after the i.v. silk spheres injection, the detailed histopathological evaluation did not show either accumulation of the silk particles, immune cell infiltration, or tissue damage in the organs. The biochemical and morphological analysis of blood confirmed that i.v. administrated spheres did not damage the internal organs and indicated that the spheres were hemocompatible, non-thrombogenic, and likely did not activate the immune system^30^. The present study aimed to investigate in detail the immunogenic properties of the MS1 and H2.1MS1 silk spheres in vivo in a mouse model.

## Results

**Figure 1:**
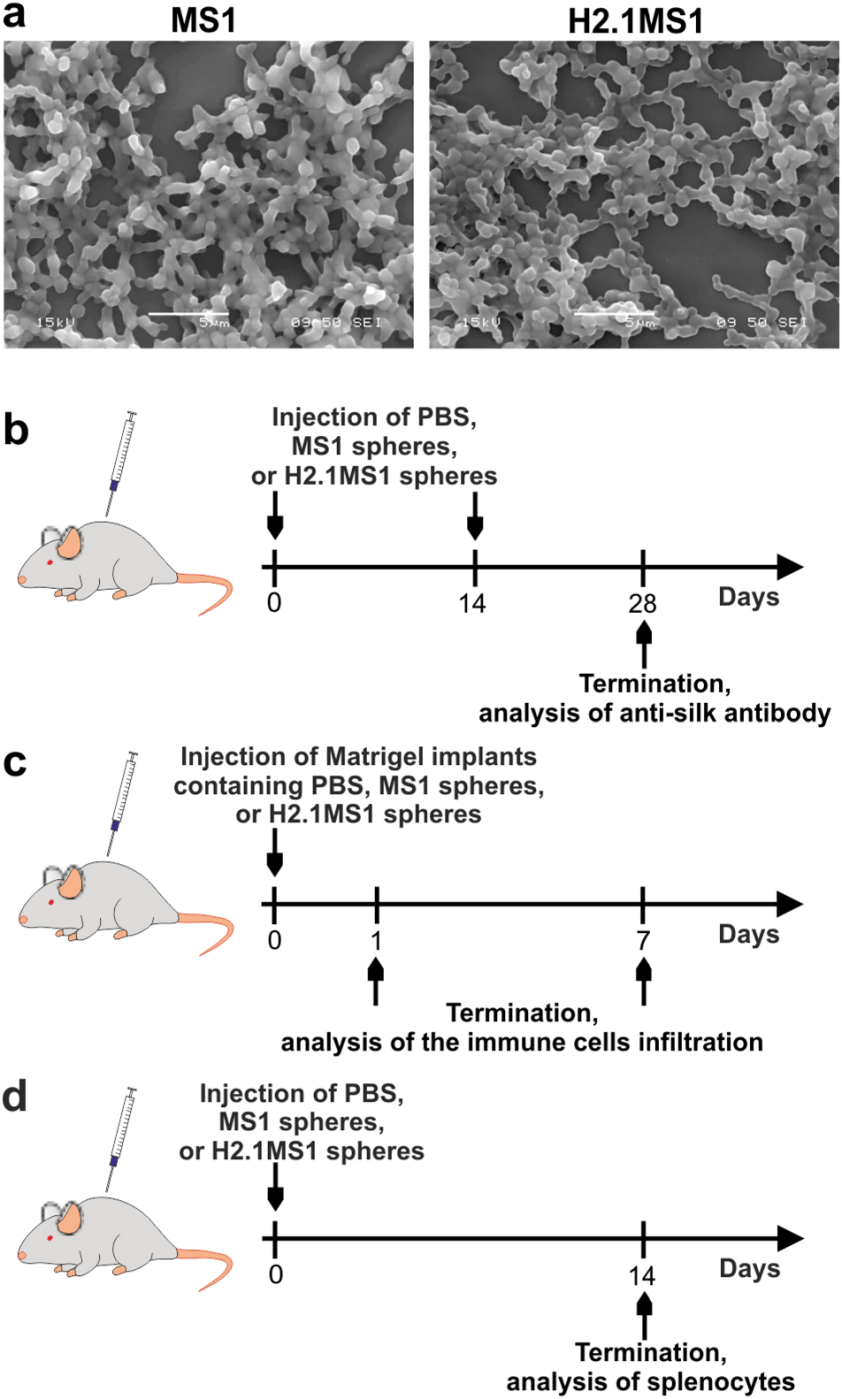
Morphology of the MS1 and H2.1MS1 spheres and a schematic representation of experiments. (**a**) SEM micrographs of the MS1 and H2.1MS1 spheres at 5000 × magnification (scale bar = 5 µm). A schematic representation of experiment for analysis of (**b**) antibody production in response to silk spheres, (**c**) immune cells infiltration at the site of silk sphere administration, and (**d**) phenotype and activity of splenocytes after silk spheres administration.

The MS1 and H2.1MS1 silk proteins were purified according to previously established protocol^26,31^. The endotoxin concentration in the silk solution was measured using the Chromogenic Endotoxin assay, which detection range is 0.1–1 EU/mL. Reliable data obtained in the range of sensitivity of the test were obtained in the presence of 5 µg of silk proteins. The endotoxin level was 0.85 EU/mL and 0.856 EU/mL for MS1 and H2.1MS1 silks, respectively. The proteins were then used to form the spheres, and the morphologies of the MS1 and H2.1MS1 spheres were confirmed by scanning electron microscopy (Fig. 1a). The immunogenicity of the silk spheres was studied in a series of experiments, and their schematic representation is shown in Fig. 1b–d.

### Specific immune response against the silk spheres

**Figure 2:**
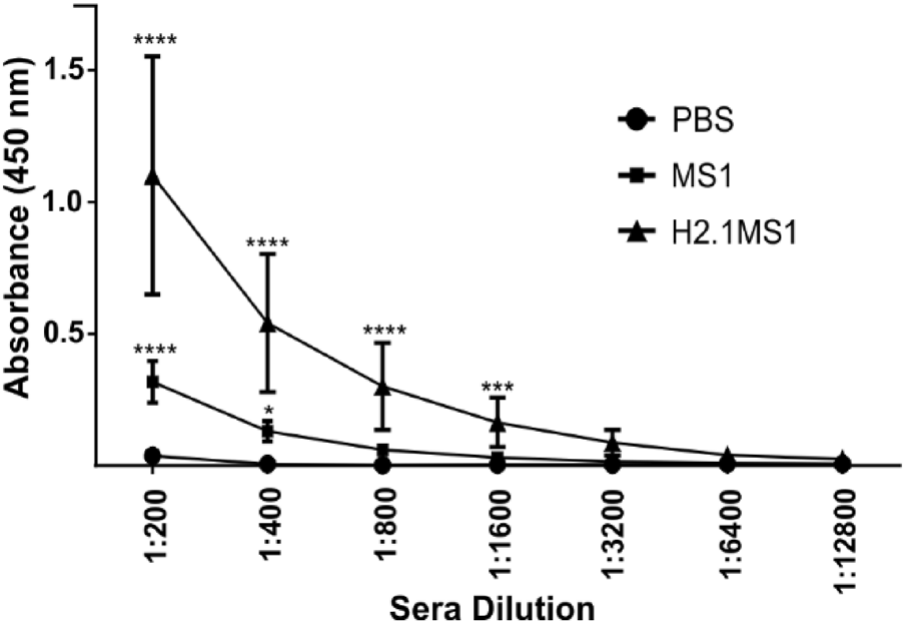
Anti-silk antibody titer in sera. Mice were injected twice with silk spheres (MS1 or H2.1MS1) or PBS. After 28 days, serum samples were collected and anti-silk antibody titers were measured using an ELISA-type assay. The results are expressed as the means ± SD for 10 animals per group. *Denotes significance at p ≤ 0.05, ***p ≤ 0.001, and ****p ≤ 0.0001**.**

Blood was collected from animals that received the MS1 or H2.1MS1 spheres twice or PBS. Specific anti-silk antibodies were detected in both tested groups (Fig. 2). In the MS1 group, the antibody titer was 400, whereas in the H2.1MS1 group, it was 1600.

### The phenotypes of the cells infiltrating the site of silk sphere administration

**Figure 3:**
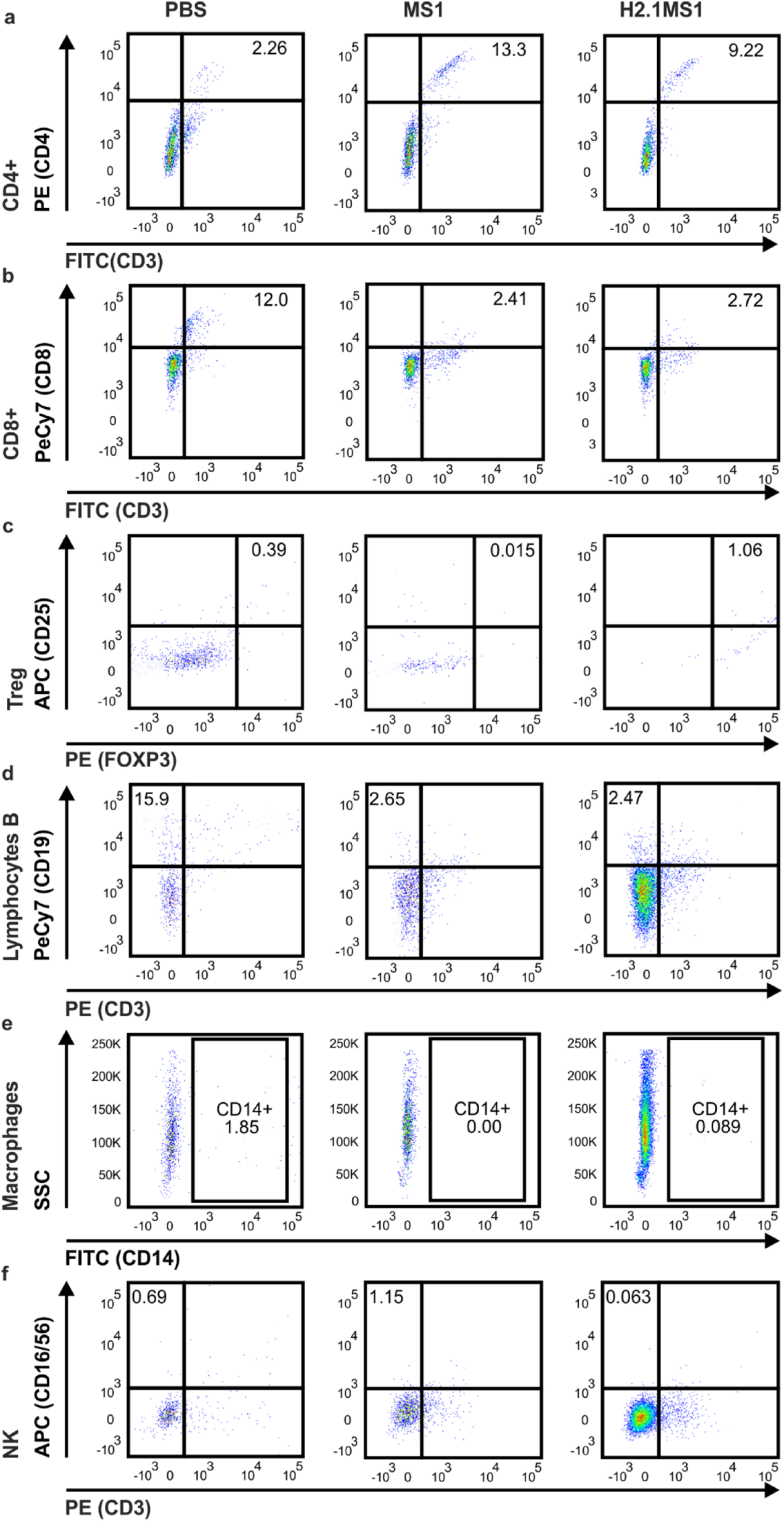
The phenotypes of the cells infiltrating the spheres 1 day after implantation. Mice were subcutaneously administered Matrigel loaded with PBS or MS1 or H2.1MS1 spheres, and 1 day after injection, the implants were excised and pooled among the groups; infiltrating immune cells were analyzed by flow cytometry. (**a**) CD4^+^ lymphocytes (CD45^+^CD3^+^CD4^+^), (**b**) CD8^+^ lymphocytes (CD45^+^CD3^+^CD8^+^), (**c**) T_reg_ lymphocytes (CD45^+^CD3^+^CD25^+^FoxP3^+^), (**d**) B lymphocytes (CD45^+^CD3^−^CD19^+^), (**e**) macrophages (CD45^+^CD14^+^), and (**f**) NK cells (CD45^+^CD3^−^CD16/56^+^).

**Figure 4:**
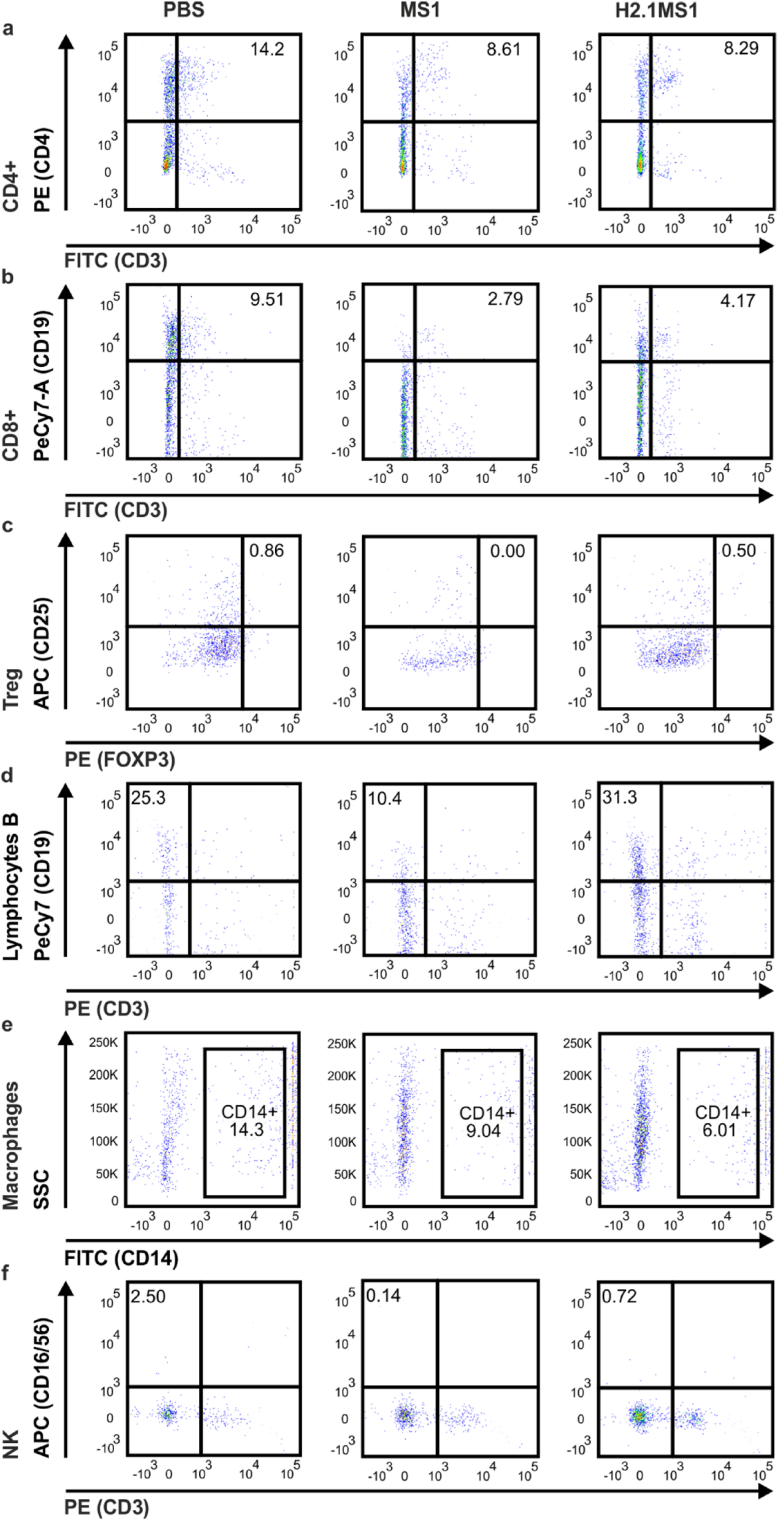
The phenotypes of the cells infiltrating the spheres 7 days after implantation. Mice were subcutaneously administered Matrigel loaded with PBS or MS1 or H2.1MS1 spheres, and 7 days after injection, the implants were excised and pooled among the groups; infiltrating immune cells were analyzed by flow cytometry. (**a**) CD4^+^ lymphocytes (CD45^+^CD3^+^CD4^+^), (**b**) CD8^+^ lymphocytes (CD45^+^CD3^+^CD8^+^), (**c**) T_reg_ lymphocytes (CD45^+^CD3^+^CD25^+^FoxP3^+^), (**d**) B lymphocytes (CD45^+^CD3^−^CD19^+^), (**e**) macrophages (CD45^+^CD14^+^), and (**f**) NK cells (CD45^+^CD3^−^CD16/56^+^).

The cells isolated from the Matrigel implants carrying PBS or the MS1 or H2.1MS1 silk spheres were analyzed cytometrically. The percentages of immune cells (CD45^+^) that infiltrated the Matrigel implants on days 1 and 7 are shown in Figs. 3 and 4, respectively. Additionally, the percentages of the cell populations at the tested time points are summarized in Table 1.

**Table 1:** The percentages of cell populations among CD45^+^ cells infiltrating the spheres.

Cell type	Day 1			Day 7		
	PBS	MS1	H2.1MS1	PBS	MS1	H2.1MS1
CD4^+^ lymphocytes	2.26	13.3	9.22	14.2	8.61	8.29
CD8^+^ lymphocytes	12	2.41	2.72	9.51	2.79	4.17
T_reg_ lymphocytes	0.39	0.015	1.06	0.86	0	0.5
Lymphocytes B	15.9	2.65	2.47	25.3	10.4	31.3
Macrophages	1.85	0	0.089	14.3	9.04	6.01
NK cells	0.69	1.15	0.063	2.5	0.14	0.72

One day after implantation, the MS1- and H2.1MS1-embedded Matrigel implants were infiltrated by a lower percentage of macrophages, CD8^+^ lymphocytes, and B lymphocytes than the control PBS group (Fig. 3). Moreover, the higher percentage of CD4^+^ lymphocytes was observed in the MS1 and H2.1MS1 group than in the control implants containing PBS. (Fig. 3). The amount of NK and Treg cells was negligible in all groups (Fig. 3).

Seven days after implantation, the most pronounced effect after administration of Matrigel-embedded samples was the increase in the percentage of macrophages and NK cells in all group, and B lymphocytes in the MS1 and H2.1MS1-carrying implants (Fig. 4). The percentage of B lymphocytes was higher in the H2.1MS1-carrying implants than the MS1 and PBS groups. Additionally, the percentage of macrophages infiltrating the H2.1MS1 implant was the lowest among the groups (Fig. 4). The percentages of CD4^+^ lymphocytes decreased in MS1 and H2.1MS1 group and was lower than in the control group. Comparing days one and seven, the percentage of CD8^+^ lymphocytes decreased in the PBS group and remained on similar level in the MS1 and H2.1MS1 samples. Treg cells remained negligible in all groups (Table 1).

### Phenotypes of the splenocytes after silk sphere administration

**Figure 5:**
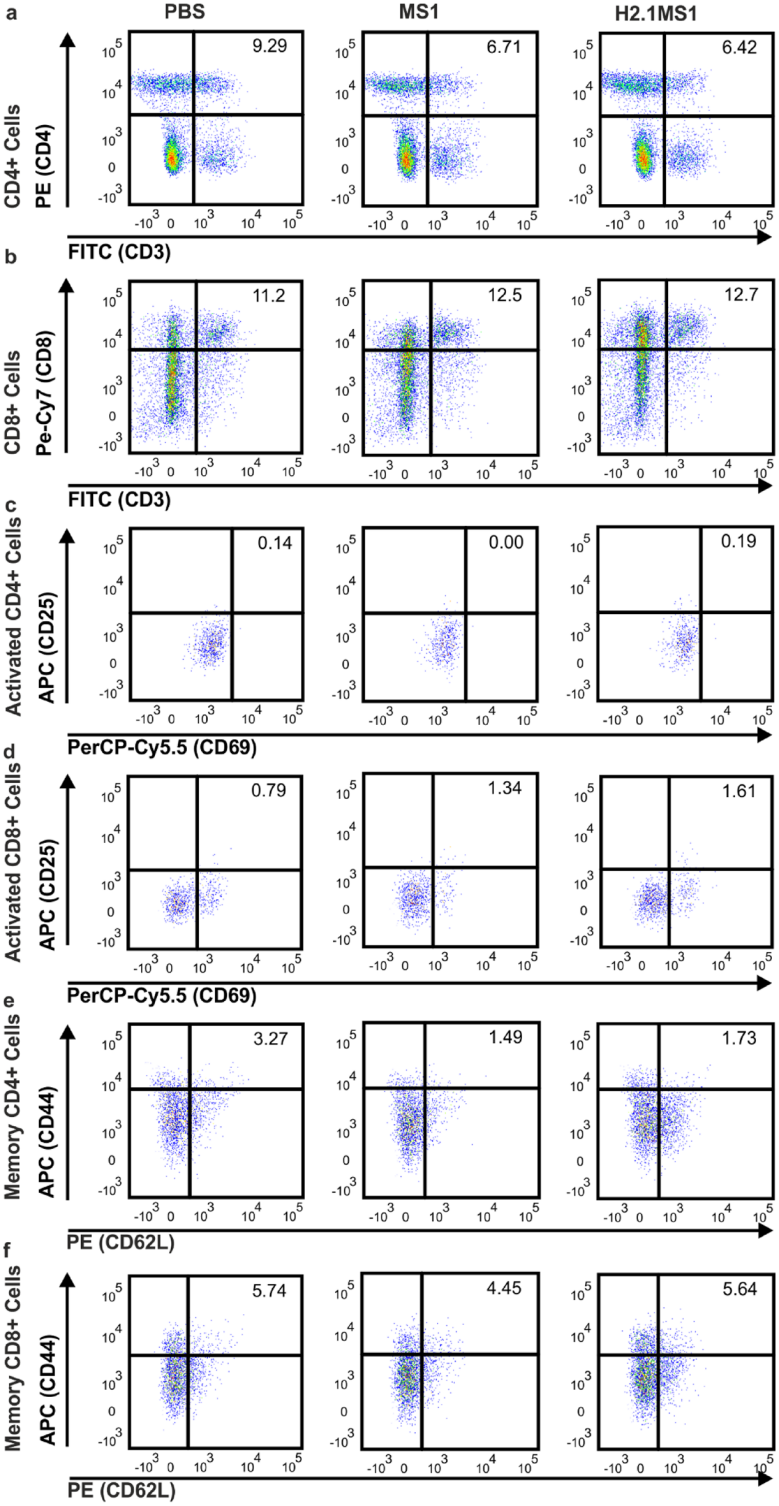
Phenotypes of splenocytes isolated from mice receiving PBS, MS1 or H2.1MS1 spheres. Mice were intravenously administered PBS, MS1, or H2.1MS1. After 14 days, the spleens were collected and pooled for each research group, and the phenotypes of the splenocytes were analyzed by flow cytometry. (**a**) CD4^+^ lymphocytes (CD3^+^CD4^+^), (**b**) CD8^+^ lymphocytes (CD3^+^CD8^+^), (**c**) activated CD4^+^ lymphocytes (CD3^+^CD4^+^CD25^+^CD69^+^), (**d**) activated CD8^+^ lymphocytes (CD3^+^CD8^+^CD25^+^CD69^+^), (**e**) memory CD4^+^ T cells (CD3^+^CD4^+^CD44^+^CD62L^+^), and (**f**) memory CD8^+^ T cells (CD3^+^CD8^+^CD44^+^CD62L^+^).

Fourteen days after intravenous administration of PBS or MS1 or H2.1MS1 spheres, the animals were euthanized, the spleens were collected and pooled among the groups, and then the splenocytes were isolated. The phenotypes of the cells were analyzed cytometrically. The results are presented in Fig. 5 and summarized in Table 2.

**Table 2:** Percentages of lymphocyte subtypes isolated from the spleens after PBS, MS1 or H2.1MS1 sphere administration.

Cell type	PBS	MS1	H2.1MS1
CD4^+^ lymphocytes	9.29	6.71	6.42
CD8^+^ lymphocytes	11.2	12.5	12.7
Activated CD4^+^ lymphocytes	0.14	0	0.19
Activated CD8^+^ lymphocytes	0.79	1.34	1.61
Memory CD4^+^ lymphocytes	3.27	1.49	1.73
Memory CD8^+^ lymphocytes	5.74	4.45	5.64

The percentage of CD4^+^ lymphocytes in PBS group was slightly elevated comparing with MS1 and H2.1MS1 groups (Fig. 5, Table 2). In both the MS1 and H2.1MS1 groups, activated CD8^+^ cells accounted for a two times higher percentage than in the control, while memory CD4^+^ cells were two times lower than in PBS group (Fig. 5, Table 2). The percentages of CD8^+^ lymphocytes, CD8^+^ memory cells, and activated CD4^+^ lymphocytes were similar in all groups (Fig. 5, Table 2).

### Proliferation of restimulated splenocytes

The isolated splenocytes (as described above) were restimulated with PBS or MS1 or H2.1MS1 spheres, and after 72 h, their proliferation rates were assessed using a BrdU assay. The obtained data are presented as the fold change of BrdU incorporation by the restimulated cells in relation to the BrdU level in unstimulated splenocytes in the given groups.

**Figure 6:**
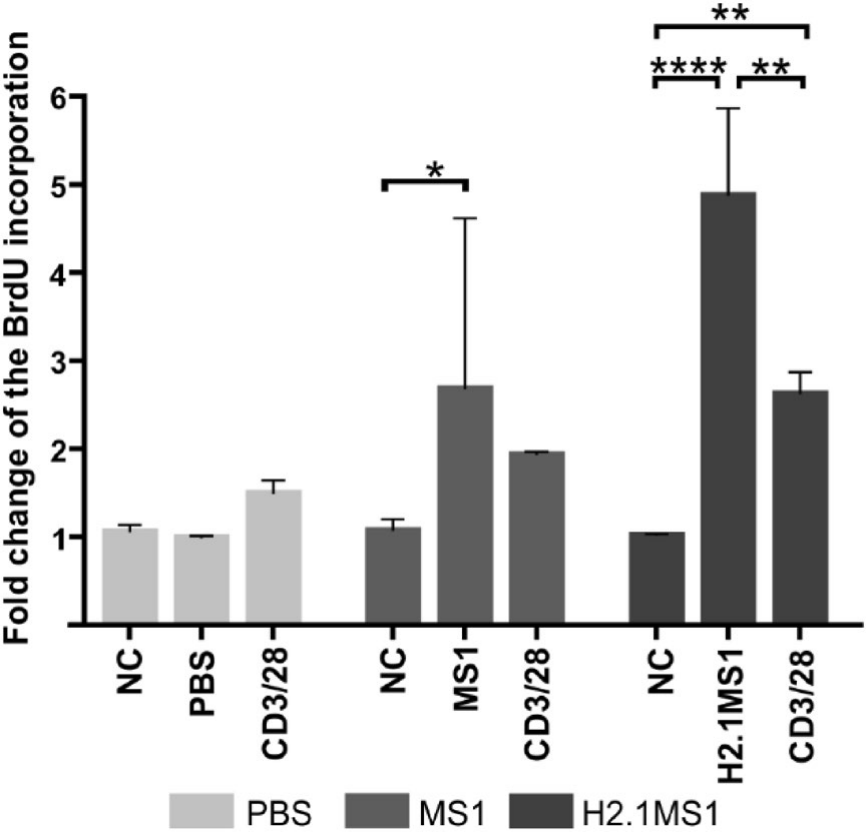
Proliferation of the restimulated splenocytes. Splenocytes were isolated from spleens 14 days after intravenous administration of PBS or MS1 or H2.1MS1 spheres. The cells were unstimulated (NC), stimulated with anti-CD3/CD28 antibodies (CD3/CD28), or accordingly challenged in vivo with PBS (PBS) or MS1 (MS1) or H2.1MS1 (H2.1MS1) particles. After 72 h, cell proliferation was measured using a BrdU assay. The results are expressed as the fold change of BrdU incorporation in relation to the corresponding control group without stimulation. The results are expressed as means ± SD. ****Indicates significance with p ≤ 0.0001, ** p ≤ 0.01, and * p ≤ 0.05.

The nonspecific stimulation of the isolated splenocytes with anti-CD3/CD28 antibodies led to higher proliferation rates of the isolated splenocytes from all groups; however, no statistical significance was observed in relation to unstimulated cells (Fig. 6). Splenocytes restimulated with the MS1 and H2.1MS1 particles proliferated significantly more than cells in the corresponding control groups (Fig. 6).

### Cytokines produced by the restimulated splenocytes

After cell restimulation and incubation for 72 h (as indicated previously), the samples were collected from above the splenocytes, and cytokine concentrations were measured using cytometric bead array.

**Figure 7:**
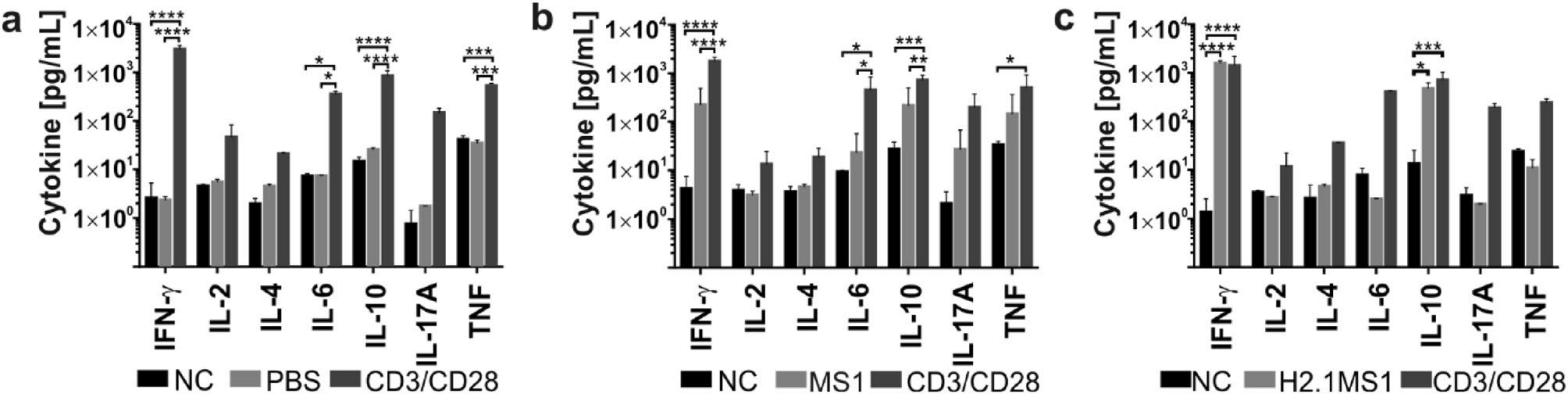
Cytokines secreted by restimulated splenocytes. Splenocytes were isolated from spleens 14 days after intravenous administration of PBS or MS1 or H2.1MS1 spheres. The cells were unstimulated (NC), stimulated with anti-CD3/CD28 antibodies (CD3/CD28), or in vivo challenge with (**a**) PBS (PBS), (**b**) MS1 spheres (MS1), or (**c**) H2.1MS1 particles (H2.1MS1). After 72 h, the cytokine concentrations in the samples collected above from the treated splenocytes were measured using cytometric bead array. The results are expressed as the means ± SD. ****Indicates significance with p ≤ 0.0001, *** p ≤ 0.001, ** p ≤ 0.01, and * p ≤ 0.05.

Cells from all tested groups responded to stimulation with anti-CD3/CD28 antibodies as the secretion of cytokines increased in relation to the unstimulated cells; in some samples, the differences were significant (Fig. 7). Although the restimulation of splenocytes with MS1 particles elevated the secretion levels of IFN-γ, IL-10, IL-17A, and TNF, the differences were not significant compared with the control samples. In contrast, restimulation with H2.1MS1 spheres led to a significant increase in IFN-γ and IL-10 concentrations compared with unstimulated cells (Fig. 7).

## Discussion

Activation of the immune system can lead to immune-related adverse effects ranging in severity from mild to life-threatening. Therefore, one of the concerns regarding the application of particle-based drug delivery systems is activation of the immune response by the used vehicles. To determine whether carriers made of the bioengineered silk proteins MS1 or H2.1MS1 can elicit an immune response in vivo, we administered these spheres to healthy mice and then analyzed (i) the phenotypes of immune cells that infiltrated the site of sphere injection, (ii) the concentrations of specific anti-silk antibodies, (iii) the percentages of lymphocyte subtypes isolated from the spleens, and (iv) splenocyte activity after ex vivo restimulation in terms of proliferation and cytokine secretion.

To study the immune cells that might potentially be attracted by silk spheres, the silk particles required immobilization within a matrix. Immobilization can be achieved by embedding the test samples within Matrigel implants. Derived from Engelbreth–Holm–Swarm mouse sarcoma, Matrigel is a mixture of various proteins (mainly laminin, collagen IV, and enactin)^32^. Additionally, as Matrigel contains various growth factors, it is not inert to cells or organisms^32,33^. Thus, it was expected that the control Matrigel plugs containing PBS would be infiltrated by immune cells. Notably, after 7 days of treatment with the PBS-containing sample, the percentages of CD4^+^ lymphocytes, macrophages, and NK cells increased profoundly. Therefore, the obtained results concerning silk sphere infiltration by immune cells were compared to implants containing PBS.

Despite the bias generated by Matrigel application, 7 days after implantation, the percentage of B lymphocytes in the H2.1MS1 group was higher than that in the PBS- and MS1-Matrigel plugs. The increased number of B lymphocytes may indicate humoral immune response formation and may be related to a higher anti-silk antibody titer than in other groups. Moreover, the restimulation of splenocytes with H2.1MS1 spheres induced the activation of lymphocytes and increased the secretion of the proinflammatory cytokine IFN-γ. Additionally, splenocytes restimulated with H2.1MS1 spheres secreted significantly more IL-10. IL-10 possesses immunosuppressive and anti-inflammatory properties. It downregulates the secretion of proinflammatory cytokines (such as IFN-γ) and helps maintain the Th1/Th2 phenotype balance, preventing tissue damage induced by uncontrollable and prolonged inflammation. This increased secretion of IL-10 might indicate a mild and temporary inflammatory reaction after exposure to the H2.1MS1 spheres. On the other hand, the higher level of IL-10 might indicate a shift in the polarization of lymphocytes from the Th1 to the Th2 phenotype and activation of the humoral immune response. This idea would correspond with higher lymphocyte B infiltration and a higher anti-silk antibody titer in the H2.1MS1 group.

The immunogenic potential of naïve MS1 silk was modest. Its administration resulted in a low titer of specific antibody production. Moreover, MS1 spheres initially attracted an elevated number of CD4^+^ lymphocytes to the injection site and increased the number of activated CD8^+^ lymphocytes in the spleen. Additionally, although ex vivo stimulation of lymphocytes with MS1 spheres did not cause a significant increase in cytokine expression, it significantly increased lymphocyte proliferation. Although the immunological response to MS1 particles was low, it should not be neglected.

The antigenicity of protein-derived pharmaceutics and drug carriers is a major concern in the drug development process. The prediction of potential B- and T-cell epitopes in the sequence of therapeutic proteins is often hard to achieve, as there are many factors that can contribute to their antigenicity. Historical methods of epitope prediction have relied on amino acid propensity scales derived from the physicochemical properties of the amino acids present in the sequence. By assigning a numerical value to each amino acid in the peptide chain, it is possible to predict (to some extent) which parts of the protein are most likely to be antigenic (based on the local average score of the sequence). Some of the proposed propensity scales were designed based on amino acid properties such as hydrophilicity (Levitt's hydrophilicity scale)^34^, antigenicity^35^, or surface accessibility^36^. Currently, many B-cell epitope prediction tools also analyze linear (i.e., continuous) epitopes of the protein, and they often combine multiple physicochemical features of the amino acid sequence. Nevertheless, this epitope prediction approach may be biased, as the protein's conformation might play an important role. The majority of the produced antibodies are specific to antigenic determinants located on the protein surface (i.e., conformational or discontinuous epitopes)^37^. Furthermore, these prediction tools often show limited success rates^38^.

Interestingly, we observed a difference in the immunogenic properties between the MS1 and H2.1MS1 groups. Both proteins have a similar silk sequence except for the addition of the H2.1 peptide to the N-terminal portion with the H2.1MS1 protein (Supplementary Fig. S1). This fact may indicate that the H2.1 peptide (MYWGDSHWLQYWYE) is more immunogenic than silk. Unfortunately, we did not investigate the properties of the H2.1 peptide alone. Nevertheless, it might be worth mentioning that the H2.1 peptide has in its sequence the antigenic amino acids mentioned by Kolaskar and Prasad, namely glutamic acid, serine and leucine^35^. Analysis from the Antibody Epitope Prediction tool available on the Immune Epitope Database and Analysis Resource website showed that according to the Kolaskar and Tongaonkar Antigenicity scale, the H2.1MS1 protein possess potential epitopes in the H2.1 peptide sequence and thus could potentially be immunogenic (Supplementary Fig. S2). This analysis also showed that potential epitopes are present in the MS1 monomer sequence, although they had lower scores (Supplementary Fig. S2). The sequence of H2.1MS1 also contains aspartic acid and glutamic acid, which are potentially immunogenic as they have high hydrophilic scores^34^. However, the analysis made by the Antibody Epitope Prediction tool with Parker Hydrophilicity Prediction showed that the sequence of H2.1 peptide had the lowest score in the whole sequence of the H2.1MS1 protein, and thus according to this prediction, it is less likely that there are B-cell epitopes (Supplementary Fig. S3). In return, according to this analysis the MS1 silk contains domains that are most likely to be antigenic (Supplementary Fig. S3).

On the other hand, the H2.1 peptide could act as an adjuvant to enhance silk antigenicity. The administration of silk spheres (MS1) led to a low immune response; however, when combined with the H2.1 peptide, a higher anti-silk antibody titer was generated. We coated the plates with the MS1 protein for the antibody titer measurements, and thus, independent of the tested silk variants, we always detected anti-silk antibodies (not directed to H2.1 peptide). Thus, the H2.1 peptide could help to present silk antigens to the immune system.

Endotoxin contamination may influence the activation of the immune system; thus, its concentration should be monitored in the tested samples. Our initial study showed no endotoxin contamination in MS1 silk solution after implementing the thermal denaturation method for its purification^31^. In this study, we examined endotoxin concentrations over a wide range of silk quantities to obtain reliable data. Indeed, the Limulus amebocyte lysate assay showed that the MS1 and H2.1MS1 proteins used to produce the spheres were not endotoxin-free. As the quantity of the endotoxin was similar for both silk samples, it does not suggest that the quantity of the endotoxin was the cause of the difference in immunogenicity between MS1 and H2.1MS1 silks. One explanation may be that the H2.1 peptide and endotoxins molecules may act together as a more potent adjuvant, contributing to higher antibody titers observed in the H2.1MS1 group. Few publications are available describing endotoxin removal from silk materials^39,40^. Unfortunately, each silk material should be examined separately as silks have significant differences in morphology and physicochemical properties. In future work, we plan to optimize the method of downstream silk purification that would allow us to reduce the endotoxin concentration in the MS1-type silk proteins.

Another factor that might contribute to the different immunogenicity of the tested silk spheres was the difference in their physicochemical properties. After intravenous administration of nanoparticles, various blood proteins can adsorb on their surfaces, resulting in the formation of a biomolecular corona^41^. Depending on the type of attached blood proteins, the different effects can be triggered in the organism. Weiss et al. indicated that the negatively charged bioengineered spider silk material derived from the ADF4 spidroin of *Araneus diadematus* [eADF4(C16)] interacted predominantly with transferrins, complement, and immunoglobulins, while a positively charged silk-variant with fibrinogen-based proteins^42^. Based on the corona composition, the negatively charged silk was predicted to elicit phagocytic uptake, complement, and inflammation responses, while the silk material with the opposite charge participated in blood clotting^42^. Both silk particles used in our study had a positive zeta potential^26^. Moreover, the zeta potential of the H2.1MS1 particles was very similar to that of the positively charged silk particles indicated in the above study by Weiss et al. (+ 15 ± 2 vs. + 15 ± 1, respectively)^26,42^. Although we applied H2.1MS1 and MS1 silk spheres intravenously to mice, no adverse effects, including blood clotting and animal death, were observed^30^. Moreover, the organismal response to the silk nanoparticles was assessed not only based on the mortality and behavior of the animals but also on the sphere biodistribution, blood morphology and biochemistry, concentrations of cytokines in the serum, and histopathological analysis of the internal organs^30^. The MS1-based spheres were systemically administered in three doses up to 20 mg/kg b.w. Since the highest dose did not cause animal death, it was chosen for further studies (including the one presented in this manuscript). Moreover, silk spheres were provided to mice multiple times (up to three times). It is worth mentioning that the analysis of cytokine concentrations (IL-6, IL-10, MCP-1, IFN-γ, TNF, and IL-12p70) did not indicate an inflammatory reaction upon exposure to silk spheres^30^.

Furthermore, both the MS1 and H2.1MS1 spheres were analyzed in vivo for their efficacy as drug delivery carriers in a Her2-positive breast cancer mouse model^29^. The MS1-type spheres carried the positively charged drug doxorubicin (Dox), which further increased their zeta potential^17^. To improve the efficacy of the therapy, the Dox dose and the frequency of treatment administration were investigated. The silk spheres were administered up to six times and in one experimental setting, at a dose higher than that in the present study (i.e., 30 mg/kg b.w.)^29^. Such treatment did not trigger systemic toxicity, as indicated by histopathological examination. Moreover, these studies were performed in immunocompetent mice, and H2.1MS1 silk spheres efficiently delivered the drug to inhibit tumor growth^29^. The Dox-loaded MS1-based spheres also did not cause death of animals due to blood clots.

In conclusion, although the silk molecule's net charge is an essential factor that may indicate a possible interaction with blood proteins, which may suggest their potential impact on the body, it is not the only parameter. The combination of size, hydrophobicity, surface charge, and roughness, among other factors, would influence the overall performance of these nanoparticles. As we described in the Introduction section, each material should be individually characterized in terms of their toxigenicity and immunogenicity. Silks are a very heterogeneous group of proteins, and each type of silk may affect the organism differently.

Silk is a material that has many potential biomedical applications. Certain examples of its uses have been proposed in research studies. Although the biodegradability, biocompatibility, mechanical properties, and possibility of producing various structural forms are features that are common among various silks, it should be noted that silks are not a homogeneous group of proteins. Nature and genetic engineering give access to silks (naïve and genetically engineered, respectively) with different amino acid sequences and thus with different physicochemical properties. Primarily, genetic engineering offers a wide range of possibilities to modify silk sequences and obtain a practically unlimited number of silk variants. Therefore, each type of silk material should be thoroughly tested for its interaction with the body, including its immunogenic potential.

The obtained results showed that the immunogenicity of the bioengineered MS1 silk is negligible in general. However, we revealed moderate activation of the immune response to the functionalized silk variant. Although intravenous administration of the H2.1MS1 spheres triggered some humoral response, it did not hamper the carrier-dependent delivery of the drug into the cancer niche. Both findings indicate that a drug delivery system based on MS1-bioengineered silk spheres has great potential for the treatment of cancer and other conditions.

## Methods

### Production and purification of silk

The silk protein MS1 and its functionalized variant H2.1MS1 were produced in an *E. coli* expression system and purified as described previously with slight modification^31^. Briefly, the *E. coli* cells BLR(DE3) (Novagen, Madison, WI) carrying plasmids encoding either MS1 or H2.1MS1 were used for large-scale expression of silk proteins in the BioFlo415 fermentor. Cells were grown to OD_600_ of approximately 10 and then induced with 1 mM isopropyl-1-thio-b-d-galactopyranoside IPTG (A&A Biotechnology, Gdansk, Poland). After additional 4 h, the bacterial biomass was collected by centrifugation (at 3500×*g*), and then the bacteria were resuspended in the buffer containing 20 mM HEPES pH 7.5 (Sigma-Aldrich, Saint Louis, MO), 100 mM NaCl (Sigma-Aldrich, Saint Louis, MO), protease inhibitor cocktail (Thermo Scientific, Waltham, MA), and 0.2 mg/mL lysozyme (Thermo Scientific, Waltham, MA). Next, the lysate was disrupted by sonication using Microson Ultrasonic Cell Disruptor XL (Misonix, Farmingdale, NY), and then treated with 0.1 mg/mL DNase I (Sigma-Aldrich, Saint Louis, MO) in the presence of 3 mM MgCl_2_ (Sigma, St. Louis, MO). Soluble bacterial proteins were precipitated by heat denaturation at 80 °C for 10 min and then were removed by sedimentation at 20,000×*g* for 30 min at 4 °C. Next, the supernatant was additionally denatured at 80 °C for 20 min, bacterial debris were removed by centrifugation, and then the soluble silk proteins in the supernatant were precipitated by adding the 20% ammonium sulfate (VWR, Radnor, PA). The pellet was rinsed with 20% ammonium sulfate and then dissolved in 6 M guanidinium thiocyanate (Sigma-Aldrich, Saint Louis, MO). The obtained protein was dialyzed against 10 mM Tris, pH 7.5 (Sigma, St. Louis, MO) using ZelluTrans Cellulose Dialysis Tubing with an MWCO of 12–14 kDa (Carl Roth, Karlsruhe, Germany). The protein concentration was determined by UV spectroscopy at 280 nm, referring to a molecular weight of 39 543 and 41 677 Da and extinction coefficient values of 22 350 and 43 320 M^−1^ cm^−1^ for MS1 and H2.1MS1 proteins, respectively. The quality of proteins was analyzed by separation on 12.5% sodium dodecyl sulfate–polyacrylamide gel electrophoresis (SDS-PAGE) gel and staining with Roti-Blue (Carl Roth, Karlsruhe, Germany).

### Endotoxin concentration measurement

The endotoxin concentration in soluble MS1 and H2.1MS1 proteins was measured with Pierce™ Chromogenic Endotoxin Quant Kit (Thermo Scientific, Waltham, MA) according to the protocol provided by the manufacturer. In brief, the protein samples were prepared at the concentration of 1 mg/mL and further diluted with endotoxin-free water provided in the kit. Next, an 50 μL of tested samples, dilutions of endotoxin standard solution, and blank control (endotoxin-free water) were added to the 96-well plate. Then, 50 μL of Limulus amebocyte lysate was added to each well, and the plate was incubated at 37 °C for 12 min. After incubation, 100 μL of the chromogenic substrate was added to each well and incubated at 37 °C for an additional 6 min. After incubation, 50 μL of 25% acetic acid was added to each well to stop the reaction. The absorbance was measured at 405 nm Victor X3 MultimodePlate Reader (PerkinElmer, Waltham, MA) controlled by PerkinElmer 2030 Workstation software (Perkin-Elmer, Waltham, MA).

### Production of silk spheres

To produce spheres, the silk was mixed with 2 M potassium phosphate buffer (pH 8) according to a previously described method^27^. Briefly, the silk solution (0.5 mg/mL) and phosphate buffer were mixed at a volumetric ratio of 1:10 using a micromixing syringe pump system (neMESYS 2600 N, Cetoni GmbH, Korbuβen, Germany). The spheres were then dialyzed against sterile deionized water using a ZelluTrans dialysis tube with a molecular weight cutoff of 12–14 kDa (Carl Roth, Karlsruhe, Germany). The concentration of the spheres was determined gravimetrically using an MYA 2.4Y microscale (Radwag, Radom, Poland).

### Sphere characterization by scanning electron microscopy (SEM)

The morphology of the spheres was analyzed by SEM. SEM imaging was performed with a JEOL JSM-6380LA (JEOL. Ltd., Tokyo, Japan) under an accelerating voltage of 15 kV.

### Animals

Animal experiments were conducted according to the guidelines of the ARRIVE, and the respective national regulations after approval by the Local Ethics Committee for Animal Research, Poznan, Poland (Approval Number 35/2014 and 72/2017). Female 8- to 12-week-old BALB/cAnNCrl mice (Charles River Laboratories International, Inc., Erkrath, Germany) were used. The mice were housed under specific pathogen-free conditions with water and food provided ad libitum*.*

### Immune cell infiltrates at the site of silk sphere administration

Eighteen mice were randomly assigned to six groups (n = 3, three groups, two time points). The MS1 and H2.1MS1 spheres were reconstituted in 50 µL of PBS and mixed with 50 µL of Matrigel (Corning, Corning, NY). Mice received spheres at a dose of 20 mg/kg b.w. Animals in the control group received PBS in Matrigel. The samples injected into the right flank of the animals were collected 1 and 7 days after implantation. The isolated Matrigel implants were incubated on ice for 30 min in Cell Recovery solution (Corning, Corning, NY) and filtered through a 70 µm filter, and then the extracted cells were pooled among the groups. After blocking with TruStain FcX (BioLegend, San Diego, CA), cells were incubated with fluorescently labeled antibodies (1 µg). The following antibodies were used: CD3-FITC (clone 17A2), CD3-PE (clone 145-2C11), CD4-PE (clone GK1.5), CD4-PeCy7 (clone RM4-5), CD8-PeCy7 (clone 53–6.7), CD14-FITC (clone rmC5-3), CD19-PeCy7 (clone 1D3), FoxP3-PE (clone MF23) (BD Biosciences Pharmingen, San Jose, CA), CD25-APC (clone PC61), CD45-PerCP/Cy5.5 (clone 30-F11) (BioLegend, San Diego, CA), and CD16/56-APC (clone 275003/809220) (R&D Systems, Inc., Minneapolis, MN). To detect FoxP3, FOXP3 Fixation/Permeabilization Buffer (BioLegend, San Diego, CA) was used. The cells were analyzed with a FACS ARIA II flow cytometer (BD Biosciences Pharmingen, San Jose, CA) and FACS Diva software (BD Biosciences Pharmingen, San Jose, CA). Graphs were prepared using FlowJo v 10.5.3 (FlowJo, LLC, Ashland, OR).

### Antibody production in response to silk spheres

Thirty mice were randomly assigned to the three groups (n = 10). The MS1 and H2.1MS1 spheres were reconstituted in 100 µL of sterile PBS and administered twice (on days 1 and 14) by retroorbital injection at a dose of 20 mg/kg b.w. Animals in the control group received PBS. After 28 days, blood samples were collected and centrifuged at 1500×*g* for 10 min to separate the serum. The analysis of anti-silk antibodies was performed using an ELISA-type immunoassay. In brief, a 96-well plate was coated with MS1 (4 µg/mL) by overnight incubation at 4 °C. After washing the plate three times with PBST and blocking with PBS + 1% gelatin (Sigma-Aldrich, Saint Louis, MO), sera were added to the wells at dilutions ranging from 1:200 to 1:12,800. The specific anti-silk antibodies were detected using HRP-conjugated anti-mouse IgG (Sigma-Aldrich, Saint Louis, MO) and TMB reagent (Bio-Rad, Hercules, CA). The reaction product was measured at 450 nm with an ELx808 Ultra Microplate Reader (Bio-Tek Instruments Inc., Winooski, VT). The antibody titer was determined as the highest serum dilution at which there was a significant difference between the absorbance in the treated and control groups.

### Analysis of immune cells isolated from the spleens

Fifteen mice were randomly assigned to the 3 groups (n = 5). The MS1 and H2.1MS1 spheres were reconstituted in 100 µL of sterile PBS and administered by retro orbital injection at a dose of 20 mg/kg b.w. Animals in the control group received PBS. After 14 days, the animals were sacrificed, and the spleens were collected. The splenic tissue was mechanically disaggregated with the Medimachine System For Automated, Mechanical Disaggregation (BD Biosciences Pharmingen, San Jose, CA). The obtained cell suspension was filtered through Filcon 70 µm filters (BD Biosciences Pharmingen, San Jose, CA) and pooled among the groups. Next, ACK buffer (ammonium-chloride-potassium buffer, Lonza, Basel, Switzerland) was added to induce erythrocyte lysis. After washing with PBS, splenocytes (1 × 10^6^) were blocked with TruStain FcX and incubated in the dark for 30 min in the presence of the appropriate antibodies (1 µg of each antibody). The following antibodies were used for staining: CD3-FITC (clone 17A2), CD4-PE (clone GK1.5), CD4-PeCy7 (clone RM4-5), CD8-PeCy7 (clone 53–6.7), and CD69-PerCP-Cy5.5 (clone H1.2F3) from BD Biosciences Pharmingen, San Jose, CA and CD25-APC (clone PC61), CD44-APC (clone IM7), and CD62L-PE (clone MEL-14) from BioLegend, San Diego, CA. After washing with PBS, cells were analyzed with a FACSARIA II flow cytometer and FACSDiva software. The obtained results were analyzed using FlowJo v 10.5.3.

### Activity of the restimulated splenocytes

The isolated splenocytes were reconstituted in X-Vivo medium (Lonza, Basel, Switzerland) and seeded into a U-shaped 96-well plate (VWR, Radnor, PA) at 1 × 10^6^ per well. Splenocytes were restimulated accordingly with PBS, MS1 or H2.1MS1 spheres (5 µg each). As a positive control, the cells were stimulated with anti-CD3/CD28 antibodies (BioLegend, San Diego, CA) (0.2 µg of each). Next, splenocytes were incubated at 37 °C in a humidified atmosphere containing 5% CO_2_ for 72 h.

### Cytokine secretion by the restimulated splenocytes

After 72 h of restimulation as indicated above, 50 µL of the medium was collected from the cell culture. The medium was analyzed using a Cytometric Bead Array Mouse Th1/Th2/Th17 kit (BD Biosciences Pharmingen, San Jose, CA) to determine the levels of IL-2, IL-4, IL-6, IL-10, IL-17A, IFN-γ, and TNF according to the manufacturer’s protocol. The cytokine concentration was measured with a FACS ARIA II flow cytometer (BD Biosciences Pharmingen, San Jose, CA) and FACS Diva v 6.1.2 software. The obtained results were then analyzed by the FCAP Array v 3.0 program. The experiment was performed once in triplicate.

### Proliferation of the restimulated splenocytes

After 72 h of restimulation as indicated above, a proliferation assay was performed by using the colorimetric Cell Proliferation ELISA BrdU kit (Sigma-Aldrich, St. Louis, MO) according to manufacturer instructions. In brief, 20 µL of 100 µM BrdU was added to the cells, and the cells were incubated for an additional 2 h. After centrifugation for 10 min at 5000×*g*, the culture medium was removed, and the cells were dried at 60 °C for 1 h and fixed for 1 h with 200 µL of Fix/Denat reagent. Then, an anti-BrdU-POD peroxidase conjugated antibody was added. After 1 h of incubation at room temperature, the cells were washed three times with PBS and incubated with 100 µL of TMB substrate for 15 min, and then the reaction was stopped by adding 50 µL of 2 M H_2_SO_4_ to each well. The sample absorbance was measured at 450 nm using an ELx808 Ultra microplate reader (Bio-Tek Instruments INC., Winooski, VT) and KCJunior v 1.41.8 software (Bio-Tek Instruments INC., Winooski, VT). The obtained data are presented as the relative cell proliferation to the proliferation of unstimulated cells within a given group. The experiment was performed once in triplicate.

### Statistics

Statistical analysis was performed using GraphPad Prism 6 v 6.07 (GraphPad Software, San Diego, CA). To analyze the significant differences between groups, analysis of variance (ANOVA) with a post hoc Bonferroni correction was applied. The differences were considered significant when p ≤ 0.05 (*). The data are expressed as means ± standard deviation (SD).

## Supplementary Information

Supplementary Figures.

## Data Availability

The datasets used and/or analyzed during the current study available from the corresponding author on reasonable request.

## References

[1] Sukhanova A (2018). Dependence of nanoparticle toxicity on their physical and chemical properties. Nanoscale Res. Lett..

[2] Halamoda-Kenzaoui B, Bremer-Hoffmann S (2018). Main trends of immune effects triggered by nanomedicines in preclinical studies. Int. J. Nanomed..

[3] Ritz S (2015). Protein corona of nanoparticles: Distinct proteins regulate the cellular uptake. Biomacromol.

[4] Lundqvist M (2008). Nanoparticle size and surface properties determine the protein corona with possible implications for biological impacts. Proc. Natl. Acad. Sci. USA.

[5] Gustafson HH, Holt-Casper D, Grainger DW, Ghandehari H (2015). Nanoparticle uptake: The phagocyte problem. Nano Today.

[6] Hara E (2012). Pharmacokinetic change of nanoparticulate formulation “Lactosome” on multiple administrations. Int. Immunopharmacol..

[7] Liao L (2014). Subchronic toxicity and immunotoxicity of MeO-PEG-poly(D, L-lactic-co- glycolic acid)-PEG-OMe triblock copolymer nanoparticles delivered intravenously into rats. Nanotechnology.

[8] Yang Q, Lai SK (2015). Anti-PEG immunity: Emergence, characteristics, and unaddressed questions. Wiley Interdiscip. Rev. Nanomed. Nanobiotechnol..

[9] Zolnik BS, González-Fernández Á, Sadrieh N, Dobrovolskaia MA (2010). Minireview: Nanoparticles and the immune system. Endocrinology.

[10] Skotland T, Iversen TG, Llorente A, Sandvig K (2022). Biodistribution, pharmacokinetics and excretion studies of intravenously injected nanoparticles and extracellular vesicles: Possibilities and challenges. Adv. Drug. Deliv. Rev..

[11] Chenthamara D (2019). Therapeutic efficacy of nanoparticles and routes of administration. Biomater. Res..

[12] Aigner TB, DeSimone E, Scheibel T (2018). Biomedical applications of recombinant silk-based materials. Adv. Mater..

[13] Seib FP, Jones GT, Rnjak-Kovacina J, Lin Y, Kaplan DL (2013). pH-dependent anticancer drug release from silk nanoparticles. Adv. Healthc. Mater..

[14] Li H (2016). Self-assembled silk fibroin nanoparticles loaded with binary drugs in the treatment of breast carcinoma. Int. J. Nanomed..

[15] Numata K, Subramanian B, Currie HA, Kaplan DL (2009). Bioengineered silk protein-based gene delivery systems. Biomaterials.

[16] Wang X, Yucel T, Lu Q, Hu X, Kaplan DL (2010). Silk nanospheres and microspheres from silk/pva blend films for drug delivery. Biomaterials.

[17] Jastrzebska K (2018). Delivery of chemotherapeutics using spheres made of bioengineered spider silks derived from MaSp1 and MaSp2 proteins. Nanomedicine.

[18] Thurber AE, Omenetto FG, Kaplan DL (2015). In vivo bioresponses to silk proteins. Biomaterials.

[19] Meinel L (2005). The inflammatory responses to silk films in vitro and in vivo. Biomaterials.

[20] Panilaitis B (2003). Macrophage responses to silk. Biomaterials.

[21] Gomes S (2013). In vivo biological responses to silk proteins functionalized with bone sialoprotein. Macromol. Biosci..

[22] Fredriksson C (2009). Tissue response to subcutaneously implanted recombinant spider silk: An in vivo study. Materials.

[23] Wang Y (2008). In vivo degradation of three-dimensional silk fibroin scaffolds. Biomaterials.

[24] Kuboyama N (2013). Silk fibroin-based scaffolds for bone regeneration. J. Biomed. Mater. Res. B.

[25] Dewair M, Baur X, Ziegler K (1985). Use of immunoblot technique for detection of human IgE and IgG antibodies to individual silk proteins. J. Allergy Clin. Immunol..

[26] Florczak A, Mackiewicz A, Dams-Kozlowska H (2014). Functionalized spider silk spheres as drug carriers for targeted cancer therapy. Biomacromol.

[27] Florczak A, Jastrzebska K, Bialas W, Mackiewicz A, Dams-Kozlowska H (2018). Optimization of spider silk sphere formation processing conditions to obtain carriers with controlled characteristics. J. Biomed. Mater. Res. A.

[28] Florczak A, Jastrzebska K, Mackiewicz A, Dams-Kozlowska H (2017). Blending two bioengineered spider silks to develop cancer targeting spheres. J. Mater. Chem. B.

[29] Florczak A (2020). Functionalized silk spheres selectively and effectively deliver a cytotoxic drug to targeted cancer cells in vivo. J. Nanobiotechnol..

[30] Deptuch T (2021). MS1-type bioengineered spider silk nanoparticles do not exhibit toxicity in an in vivo mouse model. Nanomedicine.

[31] Dams-Kozlowska H (2013). Purification and cytotoxicity of tag-free bioengineered spider silk proteins. J. Biomed. Mater. Res. A.

[32] Hughes CS, Postovit LM, Lajoie GA (2010). Matrigel: A complex protein mixture required for optimal growth of cell culture. Proteomics.

[33] Serban MA, Liu Y, Prestwich GD (2008). Effects of extracellular matrix analogues on primary human fibroblast behavior. Acta Biomater..

[34] Hopp TP, Woods KR (1981). Prediction of protein antigenic determinants from amino acid sequences. Proc. Natl. Acad. Sci. USA.

[35] Kolaskar AS, Tongaonkar PC (1990). A semi-empirical method for prediction of antigenic determinants on protein antigens. FEBS Lett..

[36] Emini EA, Hughes JV, Perlow DS, Boger J (1985). Induction of hepatitis A virus-neutralizing antibody by a virus-specific synthetic peptide. J. Virol..

[37] Sanchez-Trincado JL, Gomez-Perosanz M, Reche PA (2017). Fundamentals and methods for T- and B-cell epitope prediction. J. Immunol. Res..

[38] Blythe MJ, Flower DR (2009). Benchmarking B cell epitope prediction: Underperformance of existing methods. Protein Sci.

[39] Decker RE (2018). Method for the destruction of endotoxin in synthetic spider silk proteins. Sci. Rep..

[40] Hedhammar MY (2010). Sterilized recombinant spider silk fibers of low pyrogenicity. Biomacromol.

[41] Hadjidemetriou M, Kostarelos K (2017). Nanomedicine: Evolution of the nanoparticle corona. Nat. Nanotechnol..

[42] Weiss ACG (2020). Surface modification of spider silk particles to direct biomolecular corona formation. ACS Appl. Mater. Interfaces.

