# Supplementary material for: In vivo study of the immune response to bioengineered spider silk spheres
Source: Sci Rep. 2022 Aug 5;12:13480. doi: 10.1038/s41598-022-17637-7 (PMC9356052; doi:10.1038/s41598-022-17637-7)
Supplement: Supplementary file 1 — Supplementary Figures. [file 41598_2022_17637_MOESM1_ESM.docx]

Supplementary data

**In vivo study of the immune response to bioengineered spider silk spheres**

*Tomasz Deptuch^1,2^, Karolina Penderecka^1,2^, Mariusz Kaczmarek^1,2^, Sara Molenda^1,2,^ Hanna Dams-Kozlowska^1,2,^**

**^1^** Department of Cancer Immunology, Poznan University of Medical Sciences, 15 Garbary St, 61-866 Poznan, Poland

**^2^** Department of Diagnostics and Cancer Immunology, Greater Poland Cancer Centre, 15 Garbary St, 61-866 Poznan, Poland

***Corresponding author:**

**Hanna Dams-Kozlowska, PhD**

Poznan University of Medical Sciences,

Greater Poland Cancer Centre,

15 Garbary St.,

61-866 Poznan, Poland,

Tel.: +48 61 88 50 874,

Fax.: +48 61 85 28 502

E-mail: [hanna.dams-kozlowska@wco.pl](mailto:hanna.dams-kozlowska@wco.pl)

MAS**MYWGDSHWLQYWYE**TS(GRGGLGGQGAGAAAAAGGAGQGGYGGLGSQGTS)_15_

**Figure S1. Amino acid sequence of the H2.1MS1 protein**

The bold font indicates sequence of the functional peptide H2.1, whereas the sequence in the brackets represents MS1 monomer which is repeated 15 times in the sequence of the H2.1MS1 protein.


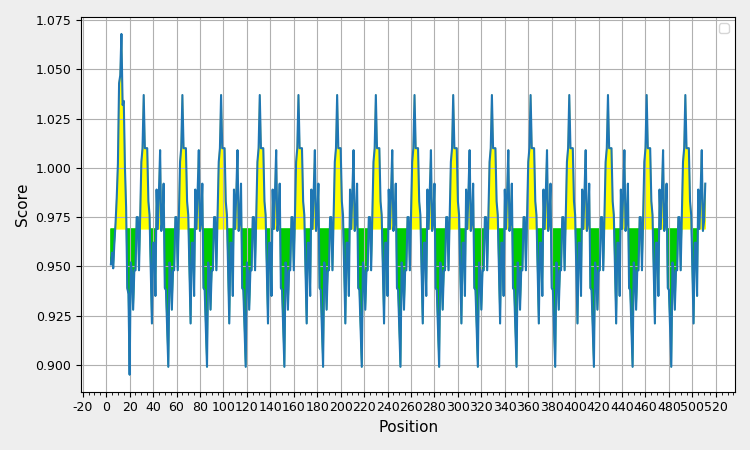


**Figure S2. Analysis of H2.1MS1 silk according to Kolaskar & Tongaonkar Antigenicity Scale.** The B-cell epitope prediction according to Kolaskar & Tongaonkar Antigenicity scale was performed with Antibody Epitope Prediction tool available on the Immune Epitope Database and Analysis Resource website (<http://tools.iedb.org/bcell/>). The x axis represents the position of amino acid in the H2.1MS1 protein sequence, whereas the y axis represents the predicted antigenic score of amino acid sequence. The fragments with high local score (yellow area under the curve) are most likely to be antigenic according to applied scale.

**
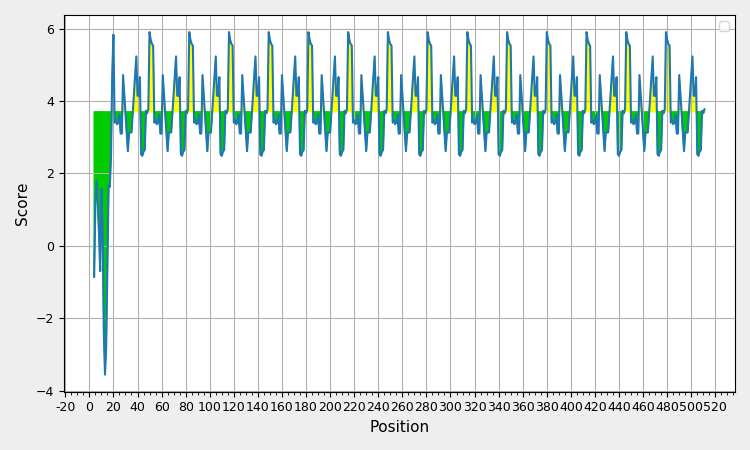
**

**Figure S3. Analysis of H2.1MS1 silk according to Parker Hydrophilicity Prediction Scale.** The B-cell epitope prediction according to Parker Hydrophilicity Prediction scale was performed with Antibody Epitope Prediction tool available on the Immune Epitope Database and Analysis Resource website (<http://tools.iedb.org/bcell/>). The x axis represents the position of amino acid in the H2.1MS1 protein sequence, whereas the y axis represents the predicted antigenic score of amino acid sequence. The fragments with high local score (yellow area under the curve) are most likely to be antigenic according to applied scale.
